# Aberrant methylation of the *Adenomatous Polyposis Coli* (*APC*) gene promoter is associated with the inflammatory breast cancer phenotype

**DOI:** 10.1038/sj.bjc.6604705

**Published:** 2008-10-07

**Authors:** I Van der Auwera, S J Van Laere, S M Van den Bosch, G G Van den Eynden, B X Trinh, P A van Dam, C G Colpaert, M van Engeland, E A Van Marck, P B Vermeulen, L Y Dirix

**Affiliations:** 1 Translational Cancer Research Group Antwerp (Lab Pathology University of Antwerp/University Hospital Antwerp, Edegem 2650, Belgium; Oncology Centre, General Hospital Sint-Augustinus, Wilrijk 2610, Belgium); 2 Department of Pathology, Research Institute GROW, University of Maastricht, Maastricht 6200 MD, The Netherlands

**Keywords:** APC, inflammatory breast cancer, laser microdissection, methylation, methylation-specific PCR

## Abstract

Aberrant methylation of the *adenomatous polyposis coli* (*APC)* gene promoter occurs in about 40% of breast tumours and has been correlated with reduced APC protein levels. To what extent epigenetic alterations of the *APC* gene may differ according to specific breast cancer phenotypes, remains to be elucidated. Our aim was to explore the role of *APC* methylation in the inflammatory breast cancer (IBC) phenotype. The status of *APC* gene promoter hypermethylation was investigated in DNA from normal breast tissues, IBC and non-IBC by both conventional and real-time quantitative methylation-specific PCR (MSP). *APC* methylation levels were compared with *APC* mRNA and protein levels. Hypermethylation of the *APC* gene promoter was present in 71% of IBC samples (*n*=21) and 43% of non-IBC samples (*n*=30) by conventional MSP (*P*=0.047). The *APC* gene also showed an increased frequency of high methylation levels in IBC (in 74% of cases, *n*=19) *vs* non-IBC (in 46% of cases, *n*=35) using a qMSP assay (*P*=0.048). We observed no significant association between *APC* methylation levels by qMSP and *APC* mRNA or protein expression levels. In conclusion, for the first time, we report the association of aberrant methylation of the *APC* gene promoter with the IBC phenotype, which might be of biological and clinical importance.

The *adenomatous polyposis coli (APC)* gene, mapped to chromosome 5q21 (Kinzler *et al*, 1991), plays a prominent role in the development of colorectal cancer, both in the autosomal dominant inherited familial *APC* syndrome (Bodmer *et al*, 1987; Groden *et al*, 1991; Joslyn *et al*, 1991; Kinzler *et al*, 1991; Nishisho *et al*, 1991) and in sporadic colorectal cancer (Fearon and Vogelstein, 1990; Miyoshi *et al*, 1992; Powell *et al*, 1992). An impaired function of APC, most often attributable to mutations within the coding sequence of the gene, leads to a lack of degradation and nuclear accumulation of *β*-catenin, which acts as a transcriptional activator, causing loss of cell growth control (Sparks *et al*, 1998). Moreover, APC functions in pathways counteracting metastasis by mediating intercellular adhesion and stabilising the cytoskeleton (Fearnhead *et al*, 2001).

Similar to findings in colorectal cancers, it has been suggested that disruption of the APC/*β*-catenin pathway may be involved in breast cancer. Loss of APC expression and upregulation of *β*-catenin have been described in human breast cancer and breast cancer cells (Ho *et al*, 1999; Jonsson *et al*, 2000; Schlosshauer *et al*, 2000). Somatic *APC* mutations are reported in only a minority of breast cancers (Furuuchi *et al*, 2000), despite high rates of allelic loss at chromosome locus 5q21 (Thompson *et al*, 1993; Medeiros *et al*, 1994). Nevertheless, epigenetic inactivation of *APC* due to DNA methylation is frequently present in both breast cancer cell lines and breast cancer tissue. In most cultured breast cancer cells, there is a complete concordance between *APC* promoter methylation and silencing of its transcript (Virmani *et al*, 2001). Cellular APC expression can be restored after demethylation with 5-aza-2′-deoxycytidine treatment. *APC* promoter methylation also occurs in a significant number of primary breast tumours (ranging from 28 to 53% of cases, depending on the applied technology) (Jin *et al*, 2001; Virmani *et al*, 2001; Liu *et al*, 2007). The frequency of *APC* methylation in primary breast tumours increases with tumour stage and size (Virmani *et al*, 2001; Roa *et al*, 2004; Chen *et al*, 2007; Liu *et al*, 2007). Moreover, hypermethylation of *APC* can be detected in breast aspirate fluid DNA (Lee *et al*, 2004) and serum DNA from patients with pre-invasive and early-stage breast cancer (Dulaimi *et al*, 2004a). DNA methylation of *APC* in serum of early breast cancer patients who had not undergone adjuvant systemic treatment appeared to be an independent prognostic marker for overall survival (Muller *et al*, 2003, 2004). These findings indicate a potential for the use of this epigenetic marker, alone or in combination with other markers, both for early detection of breast cancer and for clinical, routine risk assessment in patients with breast cancer.

Epigenetic inactivation due to hypermethylation is well established for *APC* in breast carcinoma but as yet, few studies have addressed whether epigenetic alterations of the *APC* gene might characterise specific breast cancer phenotypes. Because of their similar treatment, inflammatory breast cancer (IBC) has been rarely studied separately from other forms of locally advanced breast cancer in the past, despite differences in age-specific incidence rates, clinical presentation, histology, hormone receptor status and, finally, prognosis (Lerebours *et al*, 2005). Recent reports, however, have revealed a unique molecular profile of IBC (Bertucci *et al*, 2004; Van Laere *et al*, 2005, 2007), shedding light on its unique ability to rapidly invade and metastasise. This has led us to consider the possibility of a specific methylation pattern for this breast cancer phenotype. Notably, biological processes that are altered in IBC include cell motility, cell adhesion, regulation of the cell cycle, invasion and angiogenesis (see reference Dirix *et al*, 2006 and references therein). An important role for epigenetic silencing of *APC* in IBC might be expected based on previous studies from our group that showed low expression of this gene in IBC compared to non-IBC (unpublished data).

The aim of our study was to explore the role of *APC* methylation in the IBC phenotype. We therefore investigated independently the methylation status of the *APC* gene promoter in DNA from inflammatory and non-inflammatory breast tumours, as well as from non-neoplastic breast tissues by using both a conventional methylation-specific PCR (MSP) method and a quantitative real-time MSP (qMSP) approach. Furthermore, we compared *APC* methylation levels obtained by qMSP with APC mRNA and protein levels.

## Materials and methods

### Patients and sample collection

A total of 105 snap-frozen or paraffin-embedded human breast tumour samples was retrieved from the tissue archive of the General Hospital Sint-Augustinus, Wilrijk, Belgium. Two different data sets were used in this study (see Table 1). Conventional MSP was performed on 51 paraffin-embedded tumour samples, of which 21 were IBC and 30 were non-IBC. Patient age at diagnosis ranged from 49 to 78 years (mean, 63 years) for non-IBC patients and from 31 to 82 years (mean, 60 years) for IBC patients. In addition, 27 non-neoplastic breast tissues from women who had breast reductive surgery were used. These patient ages ranged from 24 to 62 years (mean, 42 years). qMSP was performed on 54 snap-frozen tumour samples, of which 19 were IBC and 35 were non-IBC. Patient age at diagnosis ranged from 31 to 89 years (mean, 59 years) for non-IBC patients and from 45 to 74 years (mean, 60 years) for IBC patients. In addition, 15 matched normal breast tissues adjacent to a breast tumour and 9 non-neoplastic breast tissues from women who had breast reductive surgery were used in this experiment.

All cases were randomly selected. IBC was diagnosed according to the criteria mentioned in the AJCC (American Joint Committee on Cancer)-TNM staging system (Singletary *et al*, 2002). All patients with IBC showed diffuse enlargement of the involved breast of sudden onset. There was erythema and oedema of the skin involving more than one-third of the breast. The presence of dermal lymphatic invasion as an isolated observation was not sufficient for the diagnosis of IBC and was not necessary for the diagnosis either. Relevant clinical data at diagnosis, such as age, clinical stage (AJCC TNM staging system), histological grade (Nottingham modification of the Bloom and Richardson histological grading system (Bloom and Richardson, 1957; Elston, 1984)) and hormone receptor status were obtained from medical records. Each patient gave a written informed consent. All protocols were reviewed and approved by the Ethical Committee of the General Hospital Sint-Augustinus.

### Conventional nested MSP

Paraffin sections (10 *μ*m in thickness) from the primary breast tumours were cut on a clean blade. Paraffin ribbons were then mounted on membrane-covered metal frame slides (MMI, Glattbrug, Switzerland) and dried overnight. Tissue sections were deparaffinised in xylene and rehydrated through a graded alcohol series to nuclease-free water. Tissue sections were stained with the HistoGene staining solution (Arcturus Engineering Inc., Mountain View, CA, USA), specifically designed to preserve intact nucleic acids from captured cell populations. The SL*μ*Cut system (MMI) with a solid-state UV laser was used for microdissection of tumour epithelial cells (average tissue area of 8 mm^2^).

DNA extractions from microdissected tumour epithelial cells were performed using the QIAamp DNA Micro Kit (Qiagen, Valencia, CA, USA) according to the manufacturer's protocols. Sodium bisulphite conversion of DNA samples was carried out using reagents provided in the EZ DNA Methylation Kit (Zymo Research, Orange, CA, USA). Five hundred ng of DNA were treated with sodium bisulphite following the manufacturer's recommendations.

Bisulphite-treated DNA was used as a template for a conventional nested MSP to determine the presence or absence of methylation of the promoter region of *APC*. Template amplification by a nested MSP was done essentially as described earlier (House *et al*, 2003). Step one primers flanked the CpG-rich promoter region of *APC*. PCR products of step one were diluted 1 : 1000 and subjected to the second step of MSP that incorporated a set of primers (labelled as unmethylated (U) or methylated (M)) that were designed to recognise sodium bisulphite-induced modifications of unmethylated cytosines. The primer sequences are listed elsewhere (House *et al*, 2003). Both steps of the nested MSP utilised a 25 *μ*l reaction volume, 0.5 *μ*l of Jump Start Red Taq DNA polymerase (Sigma, St Louis, MO, USA) and 4 *μ*l of DNA template. The thermal profile for step one of the nested MSP was as follows: 3 min at 95°C, then 35 repetitive cycles of denaturation (95°C × 30 s), annealing (56°C × 30 s) and extension (72°C × 30 s), followed by a final 4 min extension at 72°C. Step two of the nested MSP was performed in a similar fashion with a few adjustments to the annealing temperature (60°C) to allow for optimal template discrimination, and to the PCR cycle number (25). DNA isolated from normal peripheral lymphocytes from healthy individuals served as a negative methylation control. *In vitro* methylated DNA was used as the positive methylation control. Each PCR product (10 *μ*l) was loaded onto a 1.0% agarose gel, stained with GelStar nucleic acid stain solution (Cambrex Charles City, IA, USA) and directly visualised under UV illumination.

### Quantitative real-time MSP

DNA extractions from snap-frozen tissue specimens were performed using the QIAamp DNA Micro Kit (Qiagen, Valencia, CA, USA) according to the manufacturer's protocols. Sodium bisulphite conversion of DNA samples was carried out using the EZ DNA Methylation Kit (Zymo Research, Orange, CA, USA). Two *μ*g of DNA were treated with sodium bisulphite following the manufacturer's recommendations.

The real-time PCR-based quantification of *APC* methylation levels was done essentially as described earlier (Eads *et al*, 2000). Two sets of primers and probes, designed specifically for sodium bisulphite-converted DNA, were used: a methylated set for *APC* and a reference set, *β-actin* (*ACTB)*, to normalise for input DNA. The primers and probes used for *APC* and *ACTB* are listed elsewhere (Eads *et al*, 2000; Usadel *et al*, 2002). Fluorogenic PCRs were carried out in a reaction volume of 25 *μ*l in 96-well plates in a 7900 Sequence Detector (Applied Biosystems, Foster City, CA, USA). PCR was carried out in separate wells for each primer/probe set, and each sample was run in duplicate. The final reaction mixture consisted of 600 nmol l^−1^ of each primer (Applied Biosystems), 200 nmol l^−1^ of probe (Applied Biosystems) and 12.5 *μ*l of Universal Master Mix (Applied Biosystems). Five *μ*l of bisulphite-converted genomic DNA (250 ng) was used in each real-time MSP reaction. Thermal cycling was initiated with a first denaturation step of 95°C for 10 min. The thermal profile for the PCR was 95°C for 15 s and 60°C for 1 min. Data obtained during 50 cycles of amplification were analysed. Each plate included water blanks, a positive and a negative control. DNA isolated from normal peripheral lymphocytes from healthy individuals served as a negative methylation control. *In vitro* methylated human DNA (Zymo Research) was used as the positive methylation control.

The ratio between the values obtained in the two TaqMan analyses was used as a measure for the degree of methylation of *APC*. The percentage of fully methylated molecules at the *APC* locus was calculated by dividing the *APC/ACTB* ratio of a sample by the *APC/ACTB* ratio of fully methylated human DNA and multiplying by 100. We use the abbreviation PMR (Percentage of Methylated Reference) to indicate this measurement.

### Quantitative real-time RT–PCR

*APC* methylation levels by qMSP were compared to *APC* mRNA expression levels by qRT–PCR in 52 frozen breast tumour biopsies (of which 19 were IBC and 33 were non-IBC). For two cases, tissue was not in sufficient quantity for performing both analyses.

RNA isolation and expression analyses were performed as described earlier (Van der Auwera *et al*, 2004). Briefly, total RNA was isolated with the RNeasy Mini RNA isolation kit (Qiagen, Valencia, CA, USA) and cDNA was synthesised from 1 *μ*g of total RNA with the cDNA Archive kit (Applied Biosystems). Primers and probes were purchased from Applied Biosystems assay-on-demand. All samples were run on a 7900HT Fast Real-time PCR Detector (Applied Biosystems) in duplicate. Quantitative RT–PCR was performed in a reaction volume of 25 *μ*l including 10 *μ*l of cDNA. We averaged the expression of *ACTB* and *18SrRNA* as internal reference genes to normalise input cDNA. The 
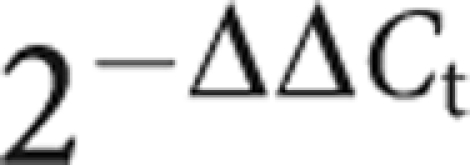
 method was used to compute relative expression values (Livak and Schmittgen, 2001).

### Immunohistochemical staining of TMA

The tissue microarray (TMA) used in this study was described earlier (Van Laere *et al*, 2007). Briefly, formalin-fixed, paraffin-embedded tissue blocks containing breast cancer were retrieved from the archives of the General Hospital St-Augustinus. Areas of invasive breast carcinoma were identified on corresponding haematoxylin and eosin-stained slides, and the tissue blocks were cored and transferred to a recipient ‘master’ block using a Tissue Microarrayer (Beecher Instruments, Silver Spring, MD, USA). A total cohort of 76 cases of breast cancer was divided between two slides. Four cores were arrayed for each specimen.

TMA slides (4 *μ*m in thickness) were deparaffinised, hydrated and subjected to antigen retrieval by heating slides in 10 mM citrate buffer (sodium citrate, pH 6.0) at 98°C for 30 min, followed by a 30 min cool-down and rinsing in wash buffer. The sections were incubated in 3% H_2_O_2_ for 10 min to inhibit endogenous peroxidase. The sections were subsequently incubated at room temperature with antibodies against the C-terminal region of the APC protein (C-20, 1 : 100, 60 min; Santa Cruz Biotechnology, Santa Cruz, CA, USA), after which sections were stained using the DAKO EnVision detection system (Dako, Glöstrup, Denmark). Sections were developed using 3,3′-diaminobenzidine for 10 min, counterstained with haematoxylin and mounted with Aquatex medium (Merck, Darmstadt, Germany).

Stained slides were then analysed for APC expression. Tumours were graded into two categories based on staining pattern: those showing loss of expression and those with expression.

### Statistical analysis

*APC* gene methylation was analysed in two ways: as a continuous variable and as a dichotomous variable (i.e., high or low, using the median PMR value as a threshold). As a continuous variable, the association of *APC* methylation with type of tumour (inflammatory *vs* non-inflammatory) and with the different groups of clinical variables was summarised using the Mann–Whitney and Kruskal–Wallis non-parametric tests, according to the number of categories. To summarise the associations between *APC* methylation (coded as high or low) and tumour type or other clinical variables we used the *χ*^2^ statistics. The Fisher's Exact Test was used when one cell had an expected count of less than 5. The level of significance was set to *P*<0.05. All analyses were carried out using SPSS version 11.0 (SPSS, Chicago, IL, USA).

## Results

### *APC* methylation analysis by conventional MSP

**Figure 1:**
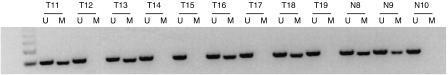
Results of conventional MSP in human breast cancer samples and normal breast tissues. The presence of a PCR product in lanes marked M indicates methylated *APC* promoter, whereas a product in lanes marked U indicates unmethylated promoter. IBC cases T11, T13 and T18, non-IBC case T16 and normal breast tissues N8 and N9 have both methylated and unmethylated promoters, whereas IBC case T17, non-IBC cases T12, T14, T15 and T19 and normal breast tissue N10 show only unmethylated promoters.

The *APC* promoter region was analysed for presence or absence of methylation in formalin-fixed and paraffin-embedded tissue samples of invasive breast cancer (including 30 cases of non-IBC and 21 cases of IBC) and of non-neoplastic breast tissue from unaffected women (*n*=27) for control purposes. Hypermethylation of the *APC* gene promoter was observed in 28 of 51 (55%) breast tumours. A low frequency of methylation (11%) was also present in non-neoplastic breast tissues (obtained from reduction mammoplasty specimens, *n*=27). When we compared methylation frequencies of breast tumours, a higher frequency of *APC* promoter methylation was found in IBC (71%) when compared to non-IBC (43%) (*P*=0.047; χ^2^). Representative examples of the MSP products of bisulphite-treated samples using primers for specific unmethylated and methylated sequences are shown in Figure 1.

It was reported earlier that gene methylation increases with age (Issa, 2000). Therefore, the high incidence of aberrant methylation in breast carcinoma specimens compared with normal breast tissue could be, at least in part, because of the older age of the average patient with breast cancer. However, a statistical analysis revealed no correlation between patient age and frequency of hypermethylation in our series of normal breast specimens (*n*=27) (*P*=0.583; Mann–Whitney test). We compared breast carcinoma specimens from patients 50 years old or younger *vs* specimens from patients older than 50 years and again, we did not observe a significant difference in frequency of *APC* methylation between these two patient groups (*P*=0.349; χ^2^).

There was a significant correlation of *APC* promoter hypermethylation with the histological grade of the tumour (*P*=0.034; χ^2^) and with tumour stage (*P*=0.026; χ^2^) in our series of breast carcinomas (*n*=51). No associations were found with other tumour characteristics (tumour size, presence of axillary metastasis, expression of ER and PR and HER2 or p53 status).

### *APC* methylation analysis by quantitative real-time MSP

A real-time MSP assay was used to quantify the relative number of methylated alleles in frozen breast tumour specimens from 35 patients with non-IBC and 19 patients with IBC. In addition, the methylation analysis was performed on DNA from matched non-neoplastic breast tissues from cancer patients (*n*=15) and from non-neoplastic breast tissues from unaffected women (*n*=9).

**Figure 2:**
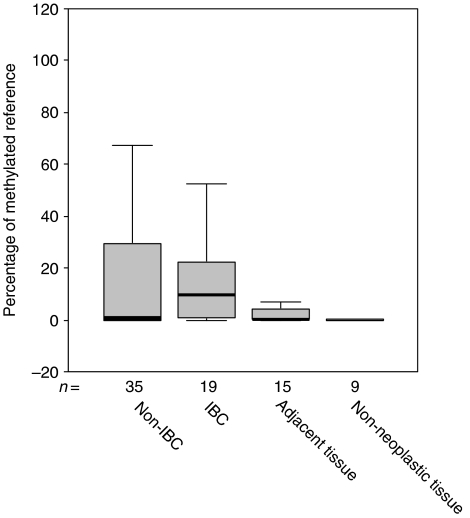
Promoter methylation levels of *APC* in non-neoplastic breast tissue (*n*=9), matched normal breast tissue adjacent to a breast tumour (*n*=15) and biopsies from patients with non-IBC (*n*=35) and patients with IBC (*n*=19). Box plots show median, upper and lower quartiles. Median PMR values were 0.03 (range, 0–0.18) for non-neoplastic breast tissue, 0.29 (range, 0–26.35) for matched normal breast tissue adjacent to a breast tumour, 0.68 (range, 0.00–94.31) for non-IBC and 9.78 (range, 0.01–68.04) for IBC.

*APC* promoter methylation was detectable in 53 of 54 (98%) of breast carcinoma samples. In seven of nine (78%) non-neoplastic breast tissues from unaffected women, *APC* promoter methylation could be detected, albeit at a very low level. The median PMR value was 0.03 (range, 0.00–0.18) for non-neoplastic breast tissue (*n*=9), 0.68 (range, 0.00–94.31) for non-IBC (*n*=35) and 9.78 (range, 0.01–68.04) for IBC (*n*=19) (Figure 2). Comparison between non-neoplastic (*n*=9) and cancer tissue (*n*=54) revealed statistically significant results (*P*=0.006; Mann–Whitney test). Comparison of PMR values between IBC and non-IBC revealed no significant results. However, when cases were coded based on methylation levels (using as a threshold the median PMR value), the frequency of IBC samples with a high *APC* methylation status (74%) was significantly increased when compared to non-IBC (46%) (*P*=0.048; χ^2^).

**Figure 3:**
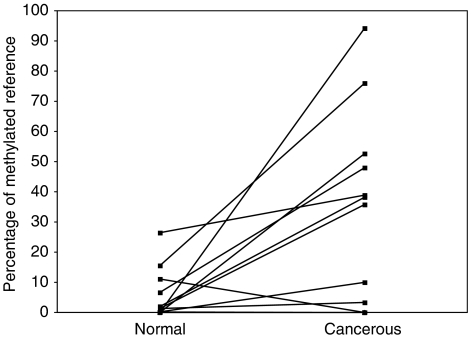
The methylation levels were compared between matched benign and breast cancer tissues from 15 patients with breast cancer. *y*-axis, the percentages of fully methylated reference in each patient sample as obtained by qMSP. *x*-axis, benign (normal) or breast cancer (cancerous) tissues.

Furthermore, we compared *APC* methylation levels in matched normal appearing breast tissue and primary breast tumour. Despite the small sample size (for only 15 of 54 patients both normal and cancerous tissue was available for analysis), we did observe a trend towards a correlation between PMR values in normal appearing adjacent tissue and carcinoma cells (*r*=0.502, *P*=0.057) (Figure 3). PMR values for *APC* were significantly elevated in tumour compared with matched normal breast tissue (*P*=0.022; Wilcoxon signed-rank test). The median level of *APC* methylation was 3.27 (range, 0.01–94.31) in tumour compared with 0.29 (range, 0–26.35) in matched normal tissue.

Overall, there were no statistical differences between PMR values in tumour tissue and clinicopathological factors (tumour size and stage, presence of axillary metastasis, histological grade, expression of ERs and HER2 or p53 status), with the exception of the expression of PR, for which a statistical trend toward higher methylation levels in PR-negative breast tumours was observed (*P*=0.075; Mann–Whitney). No association was found between PMR values in tumour tissue and patient age.

### Concordance of conventional MSP and quantitative real-time MSP

As a validation, the results of qMSP were compared with those obtained by conventional MSP in 18 patient samples (including nine cases of non-IBC and nine cases of IBC). There was a substantial agreement between results obtained by quantitative real-time MSP and those obtained by conventional MSP (*κ*=0.658, *P*=0.005). Nine of 10 cases (90%) negative for *APC* hypermethylation by conventional MSP were also negative by quantitative real-time MSP whereas six of eight cases (75%) positive for *APC* hypermethylation by conventional MSP were also positive by quantitative real-time MSP.

### Correlation of *APC* methylation levels by qMSP and *APC* mRNA expression

**Figure 4:**
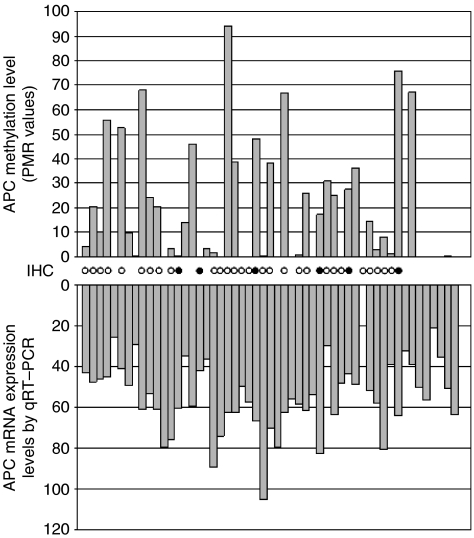
Comparison of *APC* promoter methylation, *APC* mRNA expression and APC protein expression in 54 breast tumours. (Upper) Methylation status of the *APC* locus as determined by qMSP. (Lower) *APC* mRNA expression levels measured by real-time quantitative RT–PCR (*n*=52). APC protein status is indicated by circles located between the two charts (*n*=34). An open circle indicates APC positivity, whereas a black circle denotes APC negativity, as determined by immunohistochemistry (IHC).

We compared promoter methylation status of *APC* defined by qMSP to loss of mRNA expression assessed by qRT–PCR in 52 of 54 (96%) breast tumour samples (including 19 IBC and 34 non-IBC) (Figure 4). In two breast cancer cases, frozen tissue was not sufficient in quantity for performing both analyses. The expression of *APC* appeared to be unrelated to the associated levels of *APC* methylation in breast cancer samples (*n*=52). The median *APC* mRNA expression level was 58.22 (range, 29.84–82.67) in samples with a high methylation status and 54.12 (range, 20.72–105.25) in samples with a low methylation status (*P*=0.600, Mann–Whitney).

### Correlation of *APC* methylation levels by qMSP and APC protein expression

**Figure 5:**
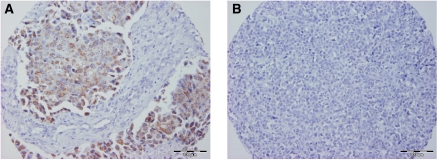
APC expression in human breast cancer. (**A**) Breast cancer with normal APC expression. (**B**) Breast cancer with loss of APC protein expression.

To determine whether *APC* promoter hypermethylation affects protein expression in breast tumours, we assessed the association between *APC* methylation levels by qMSP and loss of APC protein expression by immunohistochemistry in 34 of 54 (63%) breast tumours (including 22 cases of non-IBC and 12 cases of IBC) (Figure 4). Intensive cytoplasmic expression of the APC protein was found in 28 of 34 cases (82.3%) in our series, whereas the other six cases (17.6%) showed loss of the APC protein (Figure 5). The APC protein expression in breast cancer was independent of the *APC* methylation level. Of the 10 breast cancers with low *APC* methylation status by qMSP, eight (80%) expressed APC protein. Of the 24 cases of invasive breast cancer with high *APC* methylation status by qMSP, loss of APC protein was seen in four (16.7%).

## Discussion

Epigenetic CpG-island hypermethylation of the promoter region has been proposed as an alternative way to inactivate the *APC* tumour suppressor gene. It has been shown that *APC* gene hypermethylation can be detected in ductal carcinoma *in situ*, lobular carcinoma *in situ*, and invasive ductal and lobular tumours of all pathological grades and stages, which indicates that hypermethylation of *APC* can be a relatively early event in breast tumorigenesis (Dulaimi *et al*, 2004a). It remains to be determined to what extent epigenetic alterations of the *APC* gene might characterise specific breast cancer phenotypes. This study represents the first comprehensive comparison of the frequencies of *APC* gene promoter hypermethylation in the inflammatory and non-inflammatory breast cancer phenotype. For this purpose, the *APC* gene was analysed independently by two different techniques: a conventional MSP and a quantitative real-time MSP.

We performed laser capture microdissection of archival histological sections to collect relatively pure, or at least considerably enriched, populations of tumour epithelial cells. Because of the inherent difficulties (i.e., DNA degradation as a result of the formalin fixation and the typical small sample size) of assaying for methylation in DNA recovered from microdissected tissues, we used the nested variant of the classical MSP to analyse these samples. This technique, originally developed by Palmisano *et al* (2000), incorporates a two-stage PCR approach that allows a more sensitive (one methylated allele in >50 000 unmethylated alleles) detection of methylation in clinical samples harbouring small amounts of poor quality DNA. The frequency of *APC* methylation in our series of primary breast cancers was consistent with that recently reported by others (Jin *et al*, 2001; Virmani *et al*, 2001; Dulaimi *et al*, 2004b; Liu *et al*, 2007). Notably, specimens from patients with IBC had the highest frequency of methylation. Although the *P*-value was only just significant, IBC samples showed 1.6 times more *APC* hypermethylation than non-IBC samples. *APC* methylation was associated with histological grade and tumour stage but this should be confirmed on a larger data set.

The strictly qualitative detection of methylation by the conventional MSP is hampered by some shortcomings (Ogino *et al*, 2006): (a) conventional MSP cannot reliably distinguish low levels of methylation from high levels of methylation; (b) the frequency of hypermethylation might be overestimated (Aggerholm and Hokland, 2000; Brakensiek *et al*, 2004); and (c) assay performance characteristics are difficult to assess. To validate the acquired conventional MSP data and, also, to obtain additional information on the fraction of methylated alleles in IBC and non-IBC, we combined our conventional MSP results with those obtained by a quantitative real-time MSP assay. This assay was performed separately on DNA samples purified from frozen breast tumour biopsies collected at the time of surgery. All samples were evaluated by a pathologist to assess the presence of malignant cells. Although for only 18 samples (nine IBC and nine non-IBC), snap-frozen and formalin-fixed and paraffin-embedded biopsies were available in parallel, the quantitative real-time MSP results corroborated the observations by conventional MSP. PMR values were not significantly different in IBC and non-IBC specimens (which might reflect variation in the tumour cell content of biopsies). However, high *APC* methylation levels were 1.6 times more frequently present in IBC specimens than in non-IBC specimens, which was just statistically significant.

The methylation levels of *APC* in samples from non-neoplastic tissue adjacent to a breast tumour correlated with that observed in samples of the corresponding tumour tissue (trend for statistical significance). Notably, *APC* methylation levels in ‘normal’ tissue from cancer patients were significantly higher than in breast tissue from unaffected women and in some cases values as high as those observed in breast tumour tissue were measured. In a recent comprehensive study of methylation of *RASSF1A* promoter in breast tissue samples, it was uncovered that primary tumours had significantly higher promoter methylation than control reduction mammoplasty tissue, with adjacent normal samples having intermediate levels (Yan *et al*, 2006). Interestingly, global profiling of DNA methylation revealed more methylated genes in normal adjacent samples than in normal donor control samples. There are several possible explanations for this observation. The intermediate levels of methylation might reflect the infiltration of neoplastic cells in histologically ‘normal’ surrounding breast tissue. Alternatively, the hypermethylation of genes in samples from normal tissue adjacent to a breast tumour could be explained by field cancerisation. This concept was originally proposed by Slaughter *et al* (1953) to explain the development of multiple primary tumours and locally recurrent cancer (Slaughter *et al*, 1953). Previous studies have confirmed that genetic abnormalities exist in histologically normal breast tissues immediately adjacent to invasive cancers (Deng *et al*, 1996). Now, it has also been suggested that the primary tumour might serve as an epicentre from which methylation density progressively diffuses outwards to surrounding tissues (Yan *et al*, 2006).

We found no significant difference in *APC* mRNA and protein levels among tissues with low or high *APC* methylation status. Possible explanations for these results are: (i) samples with *APC* promoter methylation may have only one allele affected, allowing expression from the unaltered allele (Esteller *et al*, 2000); (ii) gene silencing is not a single event, but instead a series of events that begin with a marked drop in transcription and ends with its complete cessation (Turker, 2002); (iii) *APC* methylation patterns among the tumour cells that constitute a given sample might be heterogeneous and (iv) APC expression might be inactivated by gene mutations or allelic losses and not by methylation of CpG sites in the promoter region.

To conclude, we have shown that aberrant methylation of the *APC* gene promoter characterises the IBC phenotype. It should be emphasised that more investigation is required to determine to what extent the epigenetic inactivation of *APC* affects the biological behaviour of these tumours. Our study was limited not only by the small sample size, but also by the use of a candidate gene approach that was based on the observation of low gene expression. The analysis of more IBC cases and the consideration of additional genes, which is facilitated by several recently described techniques, such as methylation-sensitive arbitrarily primed PCR, restriction landmark genomic sequencing and CpG-island microarrays (see reference (Ushijima, 2005) and references therein), could present a great opportunity for enriching our knowledge of IBC biology.

## Figures and Tables

**Table 1:** Tumour characteristics

	**Conventional MSP**		**Quantitative real-time MSP**	
**Clinicopathological factors**	**IBC (*N*=21)**	**Non-IBC (*N*=30)**	**IBC (*N*=19)**	**Non-IBC (*N*=35)**
*Patients' ages (years)*				
Mean	62.7	59.8	60.0	58.6
Range	49–78	31–82	45–74	31–89
				
*Tumour stage*				
I	0	7	0	10
II	0	13	0	15
III	11	8	13	7
IV	10	2	6	3
				
*Histological type*				
Invasive ductal	21	29	16	30
Invasive lobular	0	1	3	5
				
*Histological grade*				
Well	0	5	0	11
Moderate	8	18	9	17
Poor	13	7	9	7
				
*Oestrogen receptor*				
Positive	10	18	11	30
Negative	11	12	8	5
				
*Progesterone receptor*				
Positive	7	14	4	21
Negative	14	16	15	14
